# LAYN Is a Prognostic Biomarker and Correlated With Immune Infiltrates in Gastric and Colon Cancers

**DOI:** 10.3389/fimmu.2019.00006

**Published:** 2019-01-29

**Authors:** Jing-hua Pan, Hong Zhou, Laura Cooper, Jin-lian Huang, Sheng-bin Zhu, Xiao-xu Zhao, Hui Ding, Yun-long Pan, Lijun Rong

**Affiliations:** ^1^Department of General Surgery, The First Affiliated Hospital of Jinan University, Guangzhou, China; ^2^Department of Microbiology and Immunology, College of Medicine, University of Illinois at Chicago, Chicago, IL, United States; ^3^Department of Gynecology, The First Affiliated Hospital of Jinan University, Guangzhou, China

**Keywords:** LAYN, lymphocytes, tumor-infiltrating, prognosis, gastric cancer, colon cancer

## Abstract

**Background:** Layilin (LAYN) is a critical gene that regulates T cell function. However, the correlations of LAYN to prognosis and tumor-infiltrating lymphocytes in different cancers remain unclear.

**Methods:** LAYN expression was analyzed via the Oncomine database and Tumor Immune Estimation Resource (TIMER) site. We evaluated the influence of LAYN on clinical prognosis using Kaplan-Meier plotter, the PrognoScan database and Gene Expression Profiling Interactive Analysis (GEPIA). The correlations between LAYN and cancer immune infiltrates was investigated via TIMER. In addition, correlations between LAYN expression and gene marker sets of immune infiltrates were analyzed by TIMER and GEPIA.

**Results:** A cohort (GSE17536) of colorectal cancer patients showed that high LAYN expression was associated with poorer overall survival (OS), disease-specific survival (DSS), and disease-free survival (DFS). In addition, high LAYN expression was significantly correlated with poor OS and progression-free survival (PFS) in gastric cancers (OS HR = 1.97, *P* = 3.6e-10; PFS HR = 2.12, *P* = 2.3e-10). Moreover, LAYN significantly impacts the prognosis of diverse cancers via The Cancer Genome Atlas (TCGA). Specifically, high LAYN expression was correlated with worse OS and PFS in stage 2 to 4 but not stage 1 and stage N0 gastric cancer patients (*P* = 0.28, 0.34; *P* = 0.073, 0.092). LAYN expression was positively correlated with infiltrating levels of CD4+ T and CD8+ T cells, macrophages, neutrophils, and dendritic cells (DCs) in colon adenocarcinoma (COAD) and stomach adenocarcinoma (STAD). LAYN expression showed strong correlations with diverse immune marker sets in COAD and STAD.

**Conclusions:** These findings suggest that LAYN is correlated with prognosis and immune infiltrating levels of, including those of CD8+ T cells, CD4+ T cells, macrophages, neutrophils, and DCs in multiple cancers, especially in colon and gastric cancer patients. In addition, LAYN expression potentially contributes to regulation of tumor-associated macrophages (TAMs), DCs, T cell exhaustion and Tregs in colon and gastric cancer. These findings suggest that LAYN can be used as a prognostic biomarker for determining prognosis and immune infiltration in gastric and colon cancers.

## Introduction

Gastrointestinal (GI) cancers are the most common malignancy among both men and women worldwide, and metastasis is an important biological feature that leads to a poor prognosis (1). Immune-related mechanisms play an important role in GI cancer, and immunotherapeutic strategies are considered a promising direction for the treatment of GI cancers (2). Immunotherapy, such as cytotoxic T lymphocyte associated antigen 4 (CTLA4), programmed death-1 (PD-1) and programmed death ligand-1 (PD-L1) inhibitors, showed promising antitumor effects in malignant melanoma and non-small-cell lung carcinoma (NSCLC) (3, 4). However, current immunotherapies, such as anti-CTLA4 (5, 6), showed poor clinical efficacy in metastatic colorectal and gastric cancers, anti-PD-1 and anti-PD-L1 showed a partial response in advanced colorectal and gastric cancers (7–9). In addition, an increasing number of studies have found that the tumor-infiltrating lymphocytes, such as tumor-associated macrophages (TAMs) and tumor-infiltrating neutrophils (TINs), affect the prognosis and efficacy of chemotherapy and immunotherapy (10, 11). Therefore, there is an urgent need for the elucidation of the immunophenotypes of tumor-immune interactions and identification of novel immune-related therapeutic targets in colorectal and gastric cancers.

Layilin (LAYN), which was **first** reported in 1998, is a protein encoding-gene located on chromosome 11 (12). Layilin, a 55-kDa transmembrane protein with homology to C-type lectin, is expressed in many cell types and organs. Moreover, LAYN proteins can act as a surface receptor for hyaluronan (HA). Thus, LAYN plays an important role in cell adhesion, motility, regulation of cell spreading and migration (13, 14). Previous studies (15) indicate that LAYN is involved in the tumor necrosis factor-α (TNF-α) induced epithelial-mesenchymal transition (EMT) of renal tubular epithelial cells as well as plays a critical role in HA35-induced intestinal epithelial tight junctions in inflammatory bowel disease (16). LAYN was **first** shown to be associated with cancer in a report demonstrating that low levels of LAYN protein can reduce cell invasion and lymph node metastasis A549 lung cancer cells (17). These findings suggest the LAYN plays an important role in cancer progression, invasion and metastasis.

LAYN expression is a specific signature present in colorectal cancer (CRC) and non-small cell lung changer (NSCLC) infiltrating regulatory T lymphocytes (Treg) from CRC and NSCLC patient samples. In addition, high LAYN expression was related to poor prognosis in CRC and NSCLC patients (18). A previous study (19) further found the LAYN is a crucial gene involved in liver cancer tumor-infiltrating lymphocytes. Single-cell RNA sequencing of T cells confirmed that LAYN was up regulated in activated CD8+ T and Treg cells and represses CD8+ T cell functions *in vitro*. These findings suggest that LAYN has multifaceted functional roles in Treg cells and tumor-infiltrating lymphocytes. However, the underlying functions and mechanisms of LAYN in tumor progression and tumor immunology is still unclear.

In this present study, we comprehensively analyzed LAYN expression and correlation with prognosis of cancer patients in databases such as Oncomine, PrognoScan, and Kaplan-Meier plotter. Moreover, we investigated the correlation of LAYN with tumor-infiltrating immune cells in the different tumor microenvironments via Tumor Immune Estimation Resource (TIMER). The findings in this report shed light on the important role of LAYN in colorectal and gastric cancers as well as provide a potential relationship and an underlying mechanism between LAYN and tumor-immune interactions.

## Materials and Methods

### Oncomine Database Analysis

The expression level of the LAYN gene in various types of cancers was identified in the Oncomine database (https://www.oncomine.org/resource/login.html) (20). The threshold was determined according to the following values: *P*-value of 0.001, fold change of 1.5, and gene ranking of all.

### PrognoScan Database Analysis

The correlation between LAYN expression and survival in various types of cancers was analyzed by the PrognoScan database (http://www.abren.net/PrognoScan/) (21). PrognoScan searches for relationships between gene expression and patient prognosis, such as overall survival (OS) and disease-free survival (DFS), across a large collection of publicly available cancer microarray datasets. The threshold was adjusted to a Cox *P*-value < 0.05.

### Kaplan-Meier Plotter Database Analysis

Kaplan-Meier plotter can assess the effect of 54,675 genes on survival using 10,461 cancer samples. These samples include 5,143 breast, 1,816 ovarian, 2,437 lung, and 1,065 gastric cancer samples on the HGU133 Plus 2.0 array with a mean follow-up of 69, 40, 49, and 33 months, respectively. The correlation between LAYN expression and survival in breast, ovarian, lung and gastric cancers was analyzed by Kaplan-Meier plotter (http://kmplot.com/analysis/) (22). The hazard ratio (HR) with 95% confidence intervals and log-rank *P*-value were also computed.

### TIMER Database Analysis

TIMER is a comprehensive resource for systematic analysis of immune infiltrates across diverse cancer types (https://cistrome.shinyapps.io/timer/) (23). TIMER applies a deconvolution previously published statistical method (24) to infer the abundance of tumor-infiltrating immune cells (TIICs) from gene expression profiles. The TIMER database includes 10,897 samples across 32 cancer types from The Cancer Genome Atlas (TCGA) to estimate the abundance of immune infiltrates. We analyzed LAYN expression in different types of cancer and the correlation of LAYN expression with the abundance of immune infiltrates, including B cells, CD4+ T cells, CD8+ T cells, neutrophils, macrophages, and dendritic cells, via gene modules. Gene expression levels against tumor purity is displayed on the left-most panel (25). In addition, correlations between LAYN expression and gene markers of tumor-infiltrating immune cells were explored via correlation modules. The gene markers of tumor-infiltrating immune cells included markers of CD8+ T cells, T cells (general), B cells, monocytes, TAMs, M1 macrophages, M2 macrophages, neutrophils, natural killer (NK) cells, dendritic cells (DCs), T-helper 1 (Th1) cells, T-helper 2 (Th2) cells, follicular helper T (Tfh) cells, T-helper 17 (Th17) cells, Tregs, and exhausted T cells. These gene markers are referenced in prior studies (26–28). The correlation module generated the expression scatter plots between a pair of user-defined genes in a given cancer type, together with the Spearman's correlation and the estimated statistical significance. LAYN was used for the x-axis with gene symbols, and related marker genes are represented on the y-axis as gene symbols. The gene expression level was displayed with log2 RSEM.

### Gene Correlation Analysis in GEPIA

The online database Gene Expression Profiling Interactive Analysis (GEPIA) (http://gepia.cancer-pku.cn/index.html) was used to further confirm the significantly correlated genes in TIMER. GEPIA (29) is an interactive web that includes 9,736 tumors and 8,587 normal samples from TCGA and the GTEx projects, which analyse the RNA sequencing expression. GEPIA was used to generate survival curves, including OS and DFS, based on gene expression with the log-rank test and the Mantel-Cox test in 33 different types of cancer. Gene expression correlation analysis was performed for given sets of TCGA expression data. The Spearman method was used to determine the correlation coefficient. LAYN was used for the x-axis, and other genes of interest are represented on the y-axis. The tumor and normal tissue datasets were used for analysis.

### Statistical Analysis

Survival curves were generated by the PrognoScan and Kaplan-Meier plots. The results generated in Oncomine are displayed with *P-*values, fold changes, and ranks. The results of Kaplan-Meier plots, PrognoScan, and GEPIA are displayed with HR and *P* or Cox *P*-values from a log-rank test. The correlation of gene expression was evaluated by Spearman's correlation and statistical significance, and the strength of the correlation was determined using the following guide for the absolute value: 0.00–0.19 “very weak,” 0.20–0.39 “weak,” 0.40–0.59 “moderate,” 0.60–0.79 “strong,” 0.80–1.0 “very strong.” *P*-values <0.05 were considered statistically significant.

## Results

### The mRNA Expression Levels of LAYN in Different Types of Human Cancers

**Figure 1:**
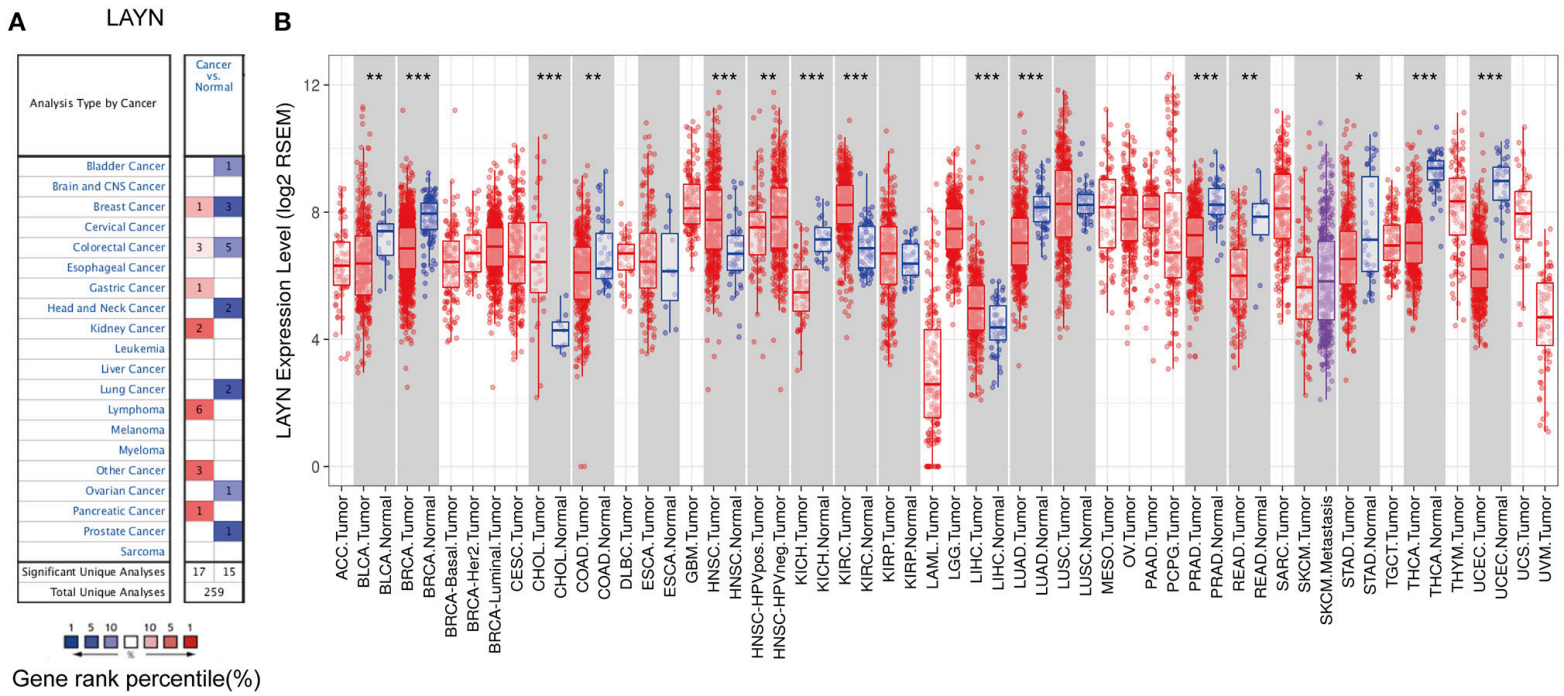
LAYN expression levels in different types of human cancers. **(A)** Increased or decreased LAYN in data sets of different cancers compared with normal tissues in the Oncomine database. **(B)** Human LAYN expression levels in different tumor types from TCGA database were determined by TIMER (^*^P < 0.05, ^**^P < 0.01, ^***^P < 0.001).

To determine differences of LAYN expression in tumor and normal tissues, the LAYN mRNA levels in different tumors and normal tissues of multiple cancer types were analyzed using the Oncomine database. This analysis revealed that the LAYN expression was higher in breast, colorectal, gastric, kidney, pancreatic cancers, and lymphoma tumors compared to the normal tissues (Figure 1A). In addition, lower expression was observed in bladder, breast, colorectal, head and neck, lung, ovarian and prostate cancers in some data sets. The detailed results of LAYN expression in different cancer types is summarized in Supplementary Table 1.

To further evaluate LAYN expression in human cancers, we examined LAYN expression using the RNA-seq data of multiple malignancies in TCGA. The differential expression between the tumor and adjacent normal tissues for LAYN across all TCGA tumors is shown in Figure 1B. LAYN expression was significantly lower in BLCA (bladder urothelial carcinoma), BRCA (breast invasive carcinoma), COAD (colon adenocarcinoma), KICH (kidney chromophobe), LUAD (lung adenocarcinoma), PRAD (prostate adenocarcinoma), READ (rectum adenocarcinoma), STAD (stomach adenocarcinoma), THCA (thyroid carcinoma), and UCEC (uterine corpus endometrial carcinoma) compared with adjacent normal tissues. However, LAYN expression was significantly higher in CHOL (cholangiocarcinoma), HNSC (head and neck cancer), KIRC (kidney renal clear cell carcinoma), and LIHC (liver hepatocellular carcinoma) compared with adjacent normal tissues.

### Prognostic Potential of LAYN in Cancers

**Figure 2:**
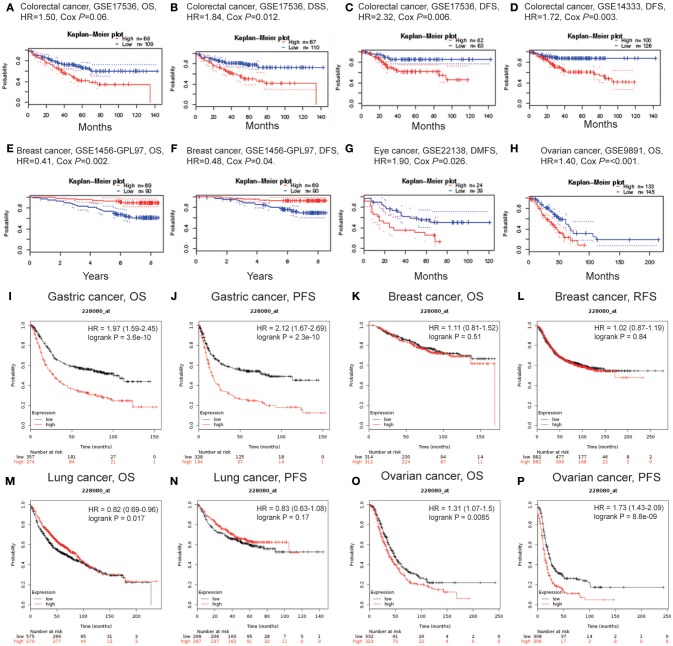
Kaplan-Meier survival curves comparing the high and low expression of LAYN in different types of cancer in the PrognoScan **(A–H)** and Kaplan-Meier plotter databases **(I–P)**. **(A–D)** Survival curves of OS, DSS, and DFS in two colorectal cancer cohorts [GSE17536 (*n* = 177) and GSE14333 (*n* = 226)]. **(E,F)** Survival curves of OS and DFS in the breast cancer cohort (GSE1456-GPL97, *n* = 159). **(G,H)** High LAYN expression was correlated with poor DMFS in the eye cancer cohort (GSE22138, *n* = 63) and poor OS in the ovarian cancer cohort (GSE9891, *n* = 278). **(I,J)** OS and DFS survival curves of gastric cancer (*n* = 631, *n* = 522). **(K,L)** OS and RFS survival curves of breast cancer (*n* = 626, *n* = 1,764). **(M,N)** OS and PFS survival curves of lung cancer (*n* = 1,145, *n* = 596). **(O,P)** OS and PFS survival curves of ovarian cancer (*n* = 655, *n* = 614). OS, overall survival; DFS, disease-free survival; RFS, relapse-free survival; DSS, disease-specific survival. DMFS, distant metastasis-free survival.

We investigated whether LAYN expression was correlated with prognosis in cancer patients. The impact of LAYN expression to survival rates was evaluated using the PrognoScan. The relationships between LAYN expression and prognosis of different cancers in PrognoScan are shown in Supplementary Table 2. Notably, LAYN expression significantly impacts prognosis in 4 type cancers, including colorectal, breast, eye and ovarian cancers (Figures 2A–H). Two cohorts (GSE17536, GSE14333) (30, 31) included 177 samples and 226 samples at different stages of colorectal cancer and showed that high LAYN expression was marginally associated with poorer prognosis (OS HR = 1.50, 95% CI = 0.98 to 2.29, Cox *P* = 0.06; DSS HR = 1.84, 95% CI=1.15 to 2.97, Cox *P* = 0.012; DFS HR = 2.32, 95% CI=1.27 to 4.25, Cox *P* = 0.006; DFS HR = 1.72, 95% CI = 1.21 to 2.46, Cox *P* = 0.003) (Figures 2A–D). Therefore, it is conceivable that high LAYN expression is an independent risk factor and leads to a poor prognosis in CRC patients.

To further examine the prognostic potential of LAYN in different cancers, Kaplan-Meier plotter database was used to evaluate the LAYN prognostic value based on Affymetrix microarrays. Interestingly, the poor prognosis in gastric (OS HR = 1.97, 95% CI = 1.59 to 2.45, *P* = 3.6e-10; PFS HR = 2.12, 95% CI = 1.67 to 2.69, *P* = 2.3e-10) and ovarian cancer (OS HR = 1.31, 95% CI = 1.07 to 1.5, *P* = 0.0085; PFS HR = 1.73, 95% CI = 1.43 to 2.09, *P* = 8.8e-9) was shown to correlate with higher LAYN expression. However, LAYN expression has less influence on breast cancer (Figures 2K,L), and shows a better OS in lung cancer (Figures 2M,N). These results suggest the LAYN expression has an impact on the prognosis of ovarian and gastric cancer (Figures 2H,I).

In addition to microarray analysis of LAYN in the PrognoScan and Kaplan-Meier plotter databases, the RNA sequencing data in TCGA were also used to analyze the prognostic potential of LAYN in different cancers via GEPIA. We analyzed relationships between LAYN expression and prognostic values in 33 types of cancer (Supplementary Figure 1). High LAYN expression levels were associated with poorer prognosis of OS and DFS in COAD, OV (ovarian serous cystadenocarcinoma), and UVM (uveal melanoma); DFS in GBM (glioblastoma multiforme) and OS in HNSC, MESO (mesothelioma) and STAD. Meanwhile, high LAYN expression was correlated with better prognosis of DFS in KIRP and THCA (thyroid carcinoma) as well as OS in SKCM (skin cutaneous melanoma). These results confirmed the prognostic value of LAYN in some specific types of cancers and that increased and decreased LAYN expression have different prognostic value depending on the type of cancer.

### High LAYN Expression Impacts the Prognosis of Gastric Cancer in Patients With Lymphatic Metastasis

To better understand the relevance and underlying mechanisms of LAYN expression in cancer, we investigated the relationship between the LAYN expression and clinical characteristics of gastric cancer patients in the Kaplan-Meier plotter databases. Overexpression of LAYN was associated with worse OS and PFS in male and female patients as well as two types of Lauren classification and differentiation (*P* < 0.05). Specifically, high LAYN mRNA expression was correlated with worse OS and PFS in stage 2 to 4 of gastric cancer patients but was not associated with OS and PFS of stage 1 (OS HR = 0.54, *P* = 0.28; PFS HR = 0.58, *P* = 0.34) and stage N0 patients (OS HR = 2.13, *P* = 0.073; PFS HR = 2.08, *P* = 0.92) (Table 1). Here the N category refers to lymph node involvement; N0 indicates no regional lymph node metastasis, and N1–N3 indicate regional lymph node metastasis (32). In addition, high LAYN expression has the highest HR values of N1 of OS and PFS in the four N categories. These results suggest that LAYN expression level can impact the prognosis in gastric cancer patient with lymph node metastasis.

**Table 1:** Correlation of LAYN mRNA expression and clinical prognosis in gastric cancer with different clinicopathological factors by Kaplan-Meier plotter.

**Clinicopathological characteristics**	**Overall survival (*****n*** **=** **882)**			**Progression-free survival (*****n*** **=** **646)**		
	***N***	**Hazard ratio**	***P-*value**	***N***	**Hazard ratio**	***P-*value**
**SEX**						
Female	187	2.7 (1.76–4.14)	**2.4e-06**	179	2.57 (1.69–3.91)	**5.1e-06**
Male	349	2.15 (1.58–2.93)	**7.4e-07**	341	2.06 (1.54–2.77)	**6.8e-07**
**STAGE**						
1	62	0.54 (0.17–1.68)	0.28	60	0.58 (0.18–1.82)	0.34
2	135	3 (1.32–6.81)	**0.0059**	131	2.32 (1.11–4.86)	**0.021**
3	197	2.15 (1.46–3.17)	**7.5e-05**	186	2.3 (1.57–3.38)	**1.1e-05**
4	140	1.96 (1.28–3)	**0.0017**	141	1.57 (1.04–2.36)	**0.029**
**STAGE T**						
2	241	1.97 (1.26–3.07)	**0.0023**	239	1.68 (1.1–2.57)	**0.016**
3	204	1.8 (1.28–2.55)	**0.00068**	204	1.74 (1.22–2.49)	**0.0021**
4	38	2.92 (1.18–7.25)	**0.016**	39	3.39 (1.44–7.95)	**0.0031**
**STAGE N**						
0	74	2.13 (0.92–4.97)	0.073	72	2.08 (0.87–4.96)	0.092
1	225	2.71 (1.75–4.2)	**3.1e-06**	222	2.38 (1.61–3.51)	**7.4e-06**
2	121	2.04 (1.27–3.26)	**0.0026**	125	2 (1.29–3.12)	**0.0018**
3	76	1.91 (1.09–3.33)	**0.021**	76	1.6 (0.93–2.76)	0.088
1+2+3	442	2.3 (1.76–3.01)	**3.4e-10**	423	2.16 (1.67–2.8)	**1.8e-09**
**STAGE M**						
0	444	2.14 (1.61–2.86)	**9.1e-08**	443	2.09 (1.6–2.73)	**2.5e-08**
1	56	1.9 (1.04–3.47)	**0.033**	56	0.7 (0.36–1.39)	**0.031**
**LAUREN CLASSIFICATION**						
Intestinal	269	2.27 (1.58–3.27)	**4.9e-06**	263	2.22 (1.55–3.19)	**7.6e-06**
Diffuse	240	2.2 (1.56–3.11)	**4.2e-06**	231	2.16 (1.53–3.06)	**8.7e-06**
**DIFFERENTIATION**						
Poor	121	1.7 (0.99–2.93)	0.053	121	1.69 (1.04–2.75)	**0.031**
Moderate	67	2.8 (1.41–5.58)	**0.0022**	67	2.89 (1.49–5.6)	**0.001**

*Bold values indicate P < 0.05*.

### LAYN Expression Is Correlated With Immune Infiltration Level in Colon and Gastric Cancers

**Figure 3:**
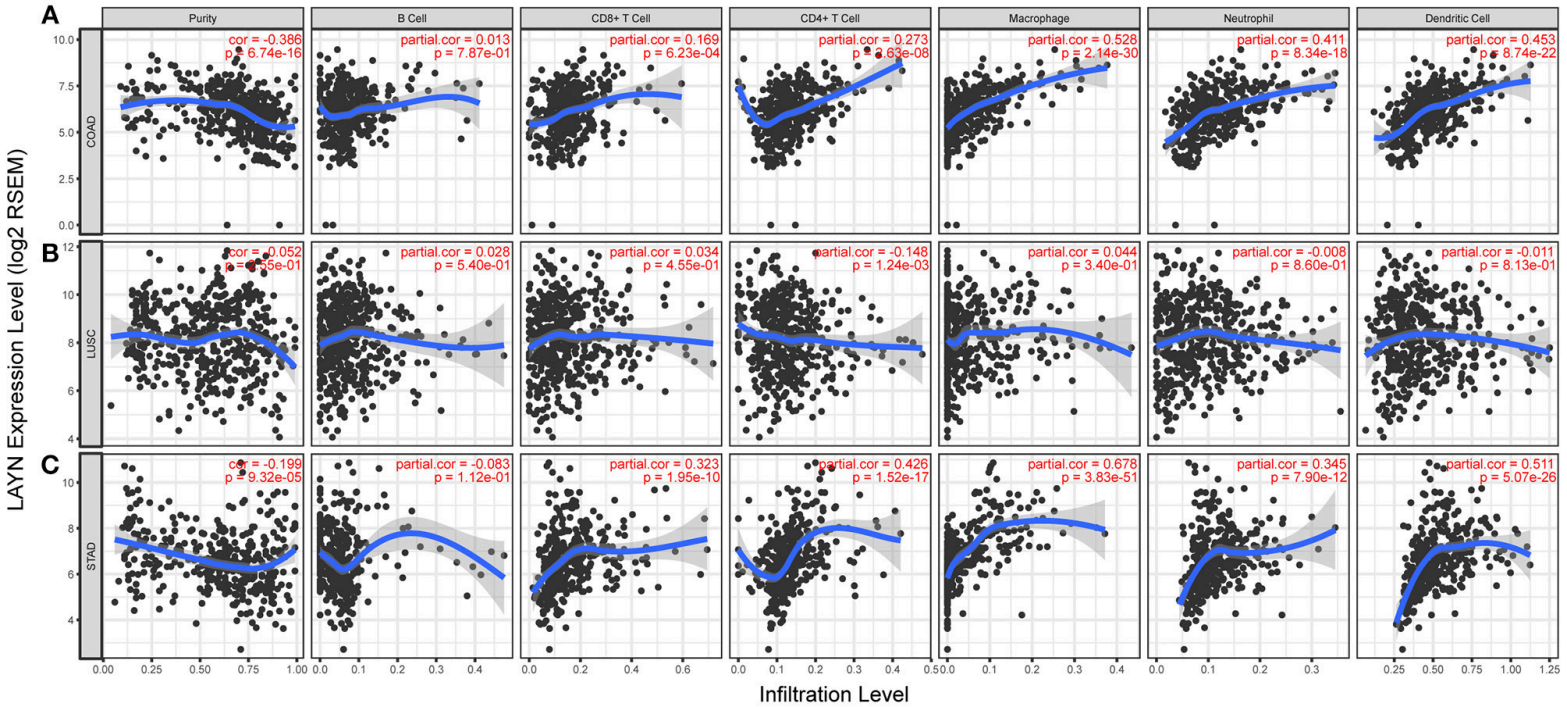
Correlation of LAYN expression with immune infiltration level in COAD (colon adenocarcinoma), LUSC (lung squamous cell carcinoma), and STAD (stomach adenocarcinoma). **(A)** LAYN expression is significantly negatively related to tumor purity and has significant positive correlations with infiltrating levels of CD8+ T cells, CD4+ T cells, macrophages, neutrophils, and dendritic cells in COAD, other than B cells (*n* = 457). **(B)** LAYN expression has no significant correlations with tumor purity and infiltrating levels of B cells, CD8+ T cells, macrophages, neutrophils, and dendritic cells in LUSC. LAYN expression showed a very weak correlation with CD4+ T cell infiltration level in LUSC (*n* = 501). **(C)** LAYN expression is significantly negatively related to tumor purity and has significant positive correlations with infiltrating levels of CD8+ T cells, CD4+ T cells, macrophages, neutrophils, and dendritic cells in STAD but no significant correlation with infiltrating level of B cells (*n* = 415).

Tumor-infiltrating lymphocytes are an independent predictor of sentinel lymph node status and survival in cancers (33, 34). Therefore, we investigated whether LAYN expression was correlated with immune infiltration levels in different types of cancer. We assessed the correlations of LAYN expression with immune infiltration levels in 39 cancer types from TIMER. The results show that LAYN expression has significant correlations with tumor purity in 26 types of cancer and significant correlations with B cell infiltration levels in 14 types of cancers. In addition, LAYN expression has significant correlations with infiltrating levels of CD8+ T cells in 18 types of cancer, CD4+ T cells in 23 types of cancer, macrophages in 23 types of cancer, neutrophils in 19 types of cancer, and dendritic cells in 25 types of cancer (Figure 3 and Supplementary Figure 2).

Given the association of LAYN expression with immune infiltration level in diverse types of cancer, we next determined the distinct types of cancers in which LAYN was associated with prognosis and immune infiltration. Tumor purity is an important factor that influences the analysis of immune infiltration in clinical tumor samples by genomic approaches (35), and TIMER and GEPIA have most of the homologous data from TCGA (23, 29). Therefore, we selected the cancer types in which LAYN expression levels have a significant negative correlation with tumor purity in TIMER and a significant correlation with prognosis in GEPIA. Interestingly, we found that LAYN expression level correlate with poorer prognosis and high immune infiltration in COAD and STAD. LAYN expression level has significant positive correlations with infiltrating levels of CD8+ T cells (*r* = 0.169, *P* = 6.23e-04), CD4+ T cells (*r* = 0.273, *P* = 2.63e-08), macrophages (*r* = 0.528, *P* = 2.14e-30), neutrophils (*r* = 0.411, *P* = 8.34e-18) and DCs (*r* = 0.453, *P* = 8.74e-22) in COAD (Figure 3A). Similarly, there were positive correlations with infiltrating levels of CD8+ T cells (*r* = 0.323, *P* = 1.95e-10), CD4+ T cells (*r* = 0.426, *P* = 1.52e-17), macrophages (*r* = 0.678, *P* = 3.83e-41), neutrophils (*r* = 0.345, *P* = 7.90e-12), and DCs (*r* = 0.551, *P* = 5.07e-26) in COAD (Figure 3C). In addtion, LAYN expression has no significant correlations with tumor purity and infiltrating levels of B cells, CD8+ T cells, macrophages, neutrophils, and dendritic cells in LUSC. These findings strongly suggest that LAYN plays a specific role in immune infiltration in colon and gastric cancers, especially those of macrophages and DCs.

### Correlation Analysis Between LAYN Expression and Immune Marker Sets

**Figure 4:**
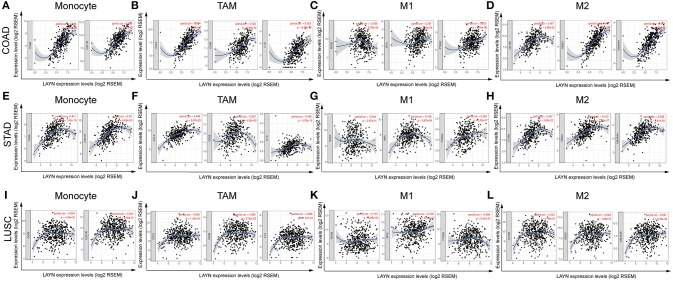
LAYN expression correlated with macrophage polarization in COAD (colon adenocarcinoma) and STAD (stomach adenocarcinoma). Markers include CD86 and CSF1R of monocytes; CCL2, CD68, and IL10 of TAMs (tumor-associated macrophages); NOS2, IRF5, and PTGS2 of M1 macrophages; and CD163, VSIG4, and MS4A4A of M2 macrophages. **(A–D)** Scatterplots of correlations between LAYN expression and gene markers of monocytes **(A)**, TAMs **(B)**, and M1 **(C)** and M2 macrophages **(D)** in COAD (*n* = 457). **(E–H)** Scatterplots of correlations between LAYN expression and gene markers of monocytes **(E)**, TAMs **(F)**, and M1 **(G)** and M2 macrophages **(H)** in STAD (*n* = 415). **(I–L)** The LUSC (lung squamous cell carcinoma) as the control group showed that LAYN expression has no significant correlation with macrophage polarization in LUSC (*n* = 501). Marker sets of macrophages in LUSC also include monocytes **(I)**, TAMs **(J)**, and M1 **(K)** and M2 macrophages **(L)**.

To investigate the relationship between LAYN and the diverse immune infiltrating cells, we focused on the correlations between LAYN and immune marker sets of various immune cells of COAD, STAD in the TIMER and GEPIA databases. We analyzed the correlations between LAYN expression and immune marker genes of different immune cells, included CD8+ T cells, T cells (general), B cells, monocytes, TAMs, M1 and M2 macrophages, neutrophils, NK cells and DCs in COAD and STAD, using lung squamous cell carcinoma (LUSC) as the control (Table 2 and Figures 4I–L). We also analyzed the different functional T cells, such as Th1 cells, Th2 cells, Tfh cells, Th17 cells, and Tregs, as well as exhausted T cells. After the correlation adjustment by purity, the results revealed the LAYN expression level was significantly correlated with most immune marker sets of various immune cells and different T cells in COAD and STAD. However, the LAYN expression level was significantly correlated with only 16 gene markers in LUSC (Table 2).

**Table 2:** Correlation analysis between LAYN and relate genes and markers of immune cells in TIMER.

**Description**	**Gene markers**	**COAD**				**STAD**				**LUSC**			
		**None**		**Purity**		**None**		**Purity**		**None**		**Purity**	
		**Cor**	***P***	**Cor**	***P***	**Cor**	***P***	**Cor**	***P***	**Cor**	***P***	**Cor**	***P***
CD8+ T cell	CD8A	0.359	^***^	0.252	^***^	0.374	^***^	0.34	^***^	−0.04	0.368	−0.062	0.174
	CD8B	0.252	^***^	0.214	^***^	0.242	^***^	0.224	^***^	−0.005	0.913	−0.022	0.627
T cell (general)	CD3D	0.335	^***^	0.22	^***^	0.324	^***^	0.281	^***^	0.000	0.998	−0.022	0.639
	CD3E	0.397	^***^	0.273	^***^	0.352	^***^	0.314	^***^	−0.023	0.608	−0.054	0.241
	CD2	0.386	^***^	0.265	^***^	0.386	^***^	0.353	^***^	0.001	0.974	−0.028	0.544
B cell	CD19	0.253	^***^	0.118	0.017	0.322	^***^	0.305	^***^	0.016	0.722	−0.006	0.896
	CD79A	0.334	^***^	0.183	^**^	0.345	^***^	0.307	^***^	0.046	0.306	0.026	0.572
Monocyte	CD86	0.697	^***^	0.646	^***^	0.468	^***^	0.441	^***^	0.068	0.127	0.034	0.464
	CD115 (CSF1R)	0.669	^***^	0.609	^***^	0.552	^***^	0.53	^***^	0.072	0.105	0.045	0.330
TAM	CCL2	0.724	^***^	0.684	^***^	0.521	^***^	0.498	^***^	0.087	0.051	0.068	0.140
	CD68	0.512	^***^	0.453	^***^	0.229	^***^	0.207	^***^	0.119	^*^	0.088	0.053
	IL10	0.553	^***^	0.523	^***^	0.445	^***^	0.436	^***^	0.08	0.074	0.066	0.147
M1 Macrophage	INOS (NOS2)	−0.198	^***^	−0.266	^***^	−0.032	0.513	−0.04	0.397	0.125	^*^	0.123	^*^
	IRF5	0.264	^***^	0.287	^***^	0.191	^***^	0.169	^**^	0.142	^*^	0.138	^*^
	COX2(PTGS2)	0.286	^***^	0.222	^***^	0.253	^***^	0.253	^***^	0.004	0.932	−0.008	0.863
M2 Macrophage	CD163	0.676	^***^	0.625	^***^	0.488	^***^	0.467	^***^	0.061	0.173	0.035	0.440
	VSIG4	0.772	^***^	0.681	^***^	0.524	^***^	0.513	^***^	.087	0.521	0.063	0.166
	MS4A4A	0.689	^***^	0.649	^***^	0.556	^***^	0.538	^***^	0.155	0.010	0.097	0.034
Neutrophils	CD66b (CEACAM8)	−0.019	0.69	0.024	0.633	−0.019	0.694	−0.01	0.868	0.049	0.271	0.038	0.402
	CD11b (ITGAM)	0.719	^***^	0.664	^***^	0.489	^***^	0.481	^***^	0.046	0.307	0.008	0.866
	CCR7	0.39	^***^	0.262	^***^	0.425	^***^	0.393	^***^	0.027	0.550	0.002	0.971
Natural killer cell	KIR2DL1	0.204	^***^	0.152	^*^	0.202	^***^	0.187	^**^	−0.135	^*^	−0.148	^*^
	KIR2DL3	0.163	^**^	0.113	0.022	0.146	^*^	0.117	0.023	−0.154	^**^	−0.17	^**^
	KIR2DL4	0.161	^**^	0.075	0.129	0.015	0.756	−0.03	0.614	−0.079	0.078	−0.105	0.022
	KIR3DL1	0.2	^***^	0.123	0.013	0.194	^***^	0.169	^**^	−0.047	0.291	−0.069	0.131
	KIR3DL2	0.188	^***^	0.096	0.052	0.199	^***^	0.166	^*^	−0.095	0.033	−0.115	0.012
	KIR3DL3	0.01	0.83	−0.011	0.833	−0.087	0.078	−0.08	0.118	−0.114	0.010	−0.13	^*^
	KIR2DS4	0.163	^**^	0.129	^*^	0.092	0.061	0.074	0.151	−0.017	0.701	−0.02	0.659
Dendritic cell	HLA-DPB1	0.596	^***^	0.52	^***^	0.377	^***^	0.329	^***^	0.043	0.338	0.023	0.622
	HLA-DQB1	0.365	^***^	0.279	^***^	0.207	^***^	0.151	^*^	0.069	0.120	0.064	0.162
	HLA-DRA	0.532	^***^	0.454	^***^	0.284	^***^	0.236	^***^	0.037	0.414	0.013	0.780
	HLA-DPA1	0.562	^***^	0.487	^***^	0.325	^***^	0.277	^***^	0.037	0.413	0.012	0.786
	BDCA-1(CD1C)	0.458	^***^	0.38	^***^	0.448	^***^	0.432	^***^	0.139	^*^	0.134	^*^
	BDCA-4(NRP1)	0.72	^***^	0.665	^***^	0.688	^***^	0.681	^***^	0.035	0.429	0.015	0.748
	CD11c (ITGAX)	0.672	^***^	0.606	^***^	0.439	^***^	0.411	^***^	0.02	0.651	−0.015	0.736
Th1	T-bet (TBX21)	0.381	^***^	0.283	^***^	0.339	^***^	0.315	^***^	−0.08	0.718	−0.111	0.016
	STAT4	0.389	^***^	0.305	^***^	0.426	^***^	0.403	^***^	0.073	0.105	0.054	0.237
	STAT1	0.351	^***^	0.292	^***^	0.041	0.41	0.012	0.811	−0.062	0.163	−0.088	0.055
	IFN-γ (IFNG)	0.218	^***^	0.17	^**^	0.081	0.1	0.005	0.334	−0.099	0.027	−0.117	0.011
	TNF-α (TNF)	0.353	^***^	0.298	^***^	0.119	0.016	0.073	0.156	0.091	0.042	0.06	0.192
Th2	GATA3	0.471	^***^	0.389	^***^	0.419	^***^	0.403	^***^	−0.08	0.074	−0.108	0.018
	STAT6	−0.103	0.03	−0.102	0.040	0.096	0.051	0.089	0.083	0.018	0.680	0.002	0.666
	STAT5A	0.211	^***^	0.179	^**^	0.404	^***^	0.388	^***^	0.006	0.899	−0.023	0.623
	IL13	0.302	^***^	0.217	^***^	0.136	^*^	0.149	^*^	−0.017	0.705	−0.039	0.400
Tfh	BCL6	0.498	^***^	0.436	^***^	0.507	^***^	0.479	^***^	0.055	0.219	0.063	0.169
	IL21	0.198	^***^	0.155	^*^	0.142	^*^	0.129	0.012	−0.084	0.059	−0.102	0.025
Th17	STAT3	0.192	^***^	0.122	0.014	0.409	^***^	0.393	^***^	0.043	0.339	0.033	0.474
	IL17A	−0.191	^***^	−0.223	^***^	−0.201	^***^	−0.21	^***^	−0.105	0.019	−0.109	0.018
Treg	FOXP3	0.553	^***^	0.46	^***^	0.319	^***^	0.282	^***^	0.038	0.395	0.007	0.878
	CCR8	0.531	^***^	0.467	^***^	0.427	^***^	0.416	^***^	0.091	0.041	0.059	0.196
	STAT5B	0.179	^**^	0.199	^***^	0.537	^***^	0.534	^***^	0.018	0.683	0.024	0.597
	TGFβ (TGFB1)	0.689	^***^	0.623	^***^	0.592	^***^	0.572	^***^	0.116	^*^	0.102	0.025
T cell exhaustion	PD-1 (PDCD1)	0.354	^***^	0.241	^***^	0.24	^***^	0.203	^***^	−0.079	0.077	−0.108	0.018
	CTLA4	0.444	^***^	0.349	^***^	0.203	^***^	0.167	^*^	0.008	0.850	−0.025	0.586
	LAG3	0.348	^***^	0.245	^***^	0.236	^***^	0.202	^***^	−0.1	0.245	−0.133	^*^
	TIM-3 (HAVCR2)	0.713	^***^	0.681	^***^	0.457	^***^	0.436	^***^	0.083	0.062	0.063	0.172
	GZMB	0.128	^*^	0.112	0.024	0.163	^**^	0.111	0.031	−0.046	0.308	−0.079	0.086

*CODA, colon adenocarcinoma; STAD, stomach adenocarcinoma; LUSC, lung squamous cell carcinoma; TAM, tumor-associated macrophage; Th, T helper cell; Tfh, Follicular helper T cell; Treg, regulatory T cell; Cor, R value of Spearman's correlation; None, correlation without adjustment. Purity, correlation adjusted by purity*.

* P < 0.01;

** P < 0.001;

*** *P < 0.0001*.

Interestingly, we found that the expression levels of most marker sets of monocytes, TAMs, M2 macrophages have strong correlations with LAYN expression in COAD and STAD (Table 2). Specifically, we showed chemokine (C-C motif) ligand (CCL)-2, CD68, IL10 of TAMs, PTGS2, IRF5 of M1 phenotype, CD163, VSIG4 and MS4A4A of M2 phenotype are significantly correlate with LAYN expression in COAD and STAD (*P* < 0.0001; Figures 4A–H). We further analyzed the correlation between LAYN expression and the above markers of monocytes and TAMs in the GEPIA database, including COAD, STAD, and LUSC. Correlation results between LAYN and markers of monocytes and TAMs are similar to those in TIMER (Table 3). These findings suggest that LAYN may regulate macrophage polarization in COAD and STAD.

**Table 3:** Correlation analysis between LAYN and relate genes and markers of monocyte and macrophages in GEPIA.

**Description**	**Gene markers**	**CODA**				**STAD**				**LUSC**			
		**Tumor**		**Normal**		**Tumor**		**Normal**		**Tumor**		**Normal**	
		**R**	***P***	**R**	***P***	**R**	***P***	**R**	***P***	**R**	***P***	**R**	***P***
Monocyte	CD86	0.75	^***^	0.074	0.65	0.5	^***^	−0.12	0.48	0.083	0.067	0.19	0.18
	CD115 (CSF1R)	0.76	^***^	−0.045	0.78	0.61	^***^	0.15	0.39	0.11	0.02	0.094	0.52
TAM	CCL2	0.8	^***^	0.53	^**^	0.55	^***^	0.62	^***^	0.11	0.014	−0.19	0.19
	CD68	0.6	^***^	−0.1	0.51	0.3	^***^	−0.49	^*^	0.14	^*^	−0.15	0.31
	IL10	0.65	^***^	0.19	0.24	0.49	^***^	0.14	0.42	0.086	0.058	0.029	0.84
M1 Macrophage	INOS (NOS2)	−0.1	0.086	−0.031	0.85	−0.002	0.98	0.1	0.56	0.15	^**^	0.17	0.22
	IRF5	0.33	^***^	−0.19	0.23	0.27	^***^	−0.33	0.049	0.18	^***^	−0.02	0.91
	COX2 (PTGS2)	0.37	^***^	0.57	^***^	0.3	^***^	0.85	^***^	0.0087	0.85	−0.14	0.33
M2 Macrophage	CD163	0.75	^***^	0.21	^***^	0.55	^***^	0.59	^**^	0.066	0.14	−0.28	0.053
	VSIG4	0.78	^***^	0.32	0.045	0.58	^***^	0.46	^*^	0.088	0.053	−0.04	0.77
	MS4A4A	0.75	^***^	0.23	0.14	0.6	^***^	0.58	^**^	0.11	0.013	0.038	0.79

*CODA, colon adenocarcinoma; STAD, stomach adenocarcinoma; LUSC, lung squamous cell carcinoma. TAM, Tumor-associated macrophages*.

*Tumor, correlation analysis in tumor tissue of TCGA. Normal, correlation analysis in normal tissue of TCGA*.

* P < 0.01;

** P < 0.001;

*** *P < 0.0001*.

High LAYN expression relates to high infiltration level of DCs in COAD and STAD, DC markers such as HLA-DPB1, BDCA-1, BDCA-4, and CD11c also show significant correlations with LAYN expression. These results further reveal that there is a strong relationship between LAYN and DCs infiltration. In addition, for Treg cells, LAYN there is a positive correlation with FOXP3 and TGFB1 in COAD and STAD. DCs can promote tumor metastasis by increasing Treg cells and reducing CD8+ T cell cytotoxicity (36). Further studies need be done on whether LAYN is a crucial factor that mediating the DC and tumor metastasis.

We also found significant correlations between LAYN and marker genes of Treg and T cell exhaustion, such as FOXP3, CCR8, STAT5B, TGFβ, PD-1, CTLA4, LAG3, and TIM-3 (Table 2). FOXP3 plays an important role in Treg cells which leads to the suppression of cytotoxic T cells attacking tumor cells (37). Interestingly, TIM-3, as a crucial gene that regulates T cell exhaustion, has a strong positive correlation with LAYN expression, suggesting that high LAYN expression plays an important role in TIM-3 mediating T cell exhaustion. Therefore, these results further confirm the findings that LAYN is specifically correlated with immune infiltrating cells in COAD and STAD which suggests that LAYN plays a vital role in immune escape in the colon and gastric cancer microenvironment.

### Discussion

LAYN is a cell surface hyaluronan (HA) receptor and it is frequently connected to cytoskeletal elements. Although LAYN has not been extensively studied, it is known that LAYN is upregulated in activated Treg and CD8+ lymphocytes. In addition, it suppresses CD8+ functions in lung, colorectal and hepatocellular cancers (18, 19). Here, we report that variations in LAYN expression level correlate to prognosis in different types of cancer. High expression level of LAYN correlates with a poorer prognosis in COAD and STAD. Interestingly, increased levels of LAYN expression can impact the prognosis of patients who have gastric cancer with lymph node metastasis indicating that LAYN expression can be used as a predictor of tumor metastasis. Furthermore, our analyses show that in colon and gastic cancers immune infiltration levels and diverse immune marker sets are correlated with levels of LAYN expression. Thus, our study provides insights in understanding the potential role of LAYN in tumor immunology and its use as a cancer biomarker.

In this study, we examined the expression levels of LAYN and systematic prognostic landscape in different types of cancers using independent datasets in Oncomine and 33 type cancers of TCGA data in GEPIA. The differential expression of LAYN between cancer and normal tissues was observed in many types of cancers. Based on the Oncomine database, we found that LAYN, compared to normal tissues, was highly expressed in breast, colorectal, gastric, kidney, pancreatic cancers, and lymphoma, while some data sets showed that LAYN has a lower level of expression in bladder, breast, colorectal, head and neck, lung, ovarian, and prostate cancers (Figure 1A). However, analysis of the TCGA data showed that LAYN expression was higher in CHOL, HNSC, KIRC, and LIHC, but lower expression in BLCA, BRCA, COAD, KICH, LUAD, PRAD, READ, STAD, THCA, and UCEC, compared with normal adjacent tissues (Figure 1B). The discrepancies in levels of LAYN expression in different cancer types in different databases may be a reflection in data collection approaches and underlying mechanisms pertinent to different biological properties. Nevertheless, in these databases we found consistent prognostic correlations between LAYN expression in colon, gastric and ovarian cancers. Analysis of the TCGA database revealed that increased LAYN expression correlated with poor prognosis in most tumor types (COAD, HNSC, MESO, OV, STAD, and UVM). KIRP, SKCM, and THCA were exeptions where high levels of LAYN expression showed a better prognosis. Furthermore, anaylsis of data from PrognoScan and Kaplan-Meier Plotter showed high level of LAYN expression was correlated with poor prognosis in colorectal, gastric and ovarian cancers. In three datasets of PrognoScan, high LAYN expression levels can be used as an independent risk factor for poor prognosis in colorectal and ovarian cancers (Figures 2A–D,H). Kaplan-Meier Plotter also showed a high LAYN expression correlated with high hazard ratio (HR) for poor overall survival (OS) and progress free survival (PFS) of gastric and ovarian cancers (Figures 2I,J,O,P). In addition, high level of LAYN expression was shown to be correlated with poor prognosis of gastric cancer in stage 2 to 4, T2 to T4 and N1 to N3, with the highest HR for poor OS and PFS when LAYN was highly expressed in gastric cancer (Table 1). Together these findings strongly suggest that LAYN is a prognostic biomarker in colorectal, gastric, and ovarian cancer.

Another important aspect of this study is that LAYN expression is correlated with diverse immune infiltration levels in cancer, especially in gastric and colon cancers. Our results demonstrate that there is a moderate to strong positive relationships between LAYN expression level and infiltration level of macrophages and DCs, and significantly positive correlations between infiltration level of CD8+ T, CD4+ T cells and neutrophils and LAYN expression in COAD and STAD (Figures 3A,C). Moreover, the correlation between LAYN expression and the marker genes of immune cells implicate the role of LAYN in regulating tumor immunology in COAD and STAD. First, gene markers of M1 macrophages such as PTGS2 and IRF5 showed weak correlations with LAYN expression, whereas M2 macrophage markers such as CD163, VSIG4, and MS4A4A showed moderate and strong correlations (Tables 2, 3). These results reveal the potential regulating role of LAYN in polarization of tumor-associated macrophages (TAM). In addition, our results indicated that LAYN has the potential to activate Tregs and induce T cell exhaustion. The increase in LAYN expression positively correlates with the expression of Treg and T cell exhaustion markers (FOXP3, CCR8, STAT5B, TIM-3, PD-1, CTLA4, and LAG3 in COAD and STAD Table 2). TIM-3, a crucial surface protein on exhausted T cells (38), is highly correlated with LAYN expression in COAD and STAD. Furthermore, significant correlations can be found between LAYN expression and the regulation of several markers of T helper cells (Th1, Th2, Tfh, and Th17) in COAD and STAD. These correlations could be indicative of a potential mechanism where LAYN regulates T cell functions in COAD and STAD. Together these findings suggest that the LAYN plays an important role in recruitment and regulation of immune infiltrating cells in COAD and STAD.

Recent studies provide possible mechanisms which explains why LAYN expression correlates with immune infiltration and poor prognosis. HA fragments act as ligands for the LAYN receptor, and LAYN is only activated when bound to HA oligosaccharides (HA fragments with low molecular weight <70 kDa) (39). HA fragments can be synthesized by tumor cells and accumulate in the tumor stroma (40). Accumulation of small HA oligosaccharides in interstitial fluid correlates with lymphatic invasion and lymph node metastasis in colorectal cancer (41). HA oligosaccharides activation of LAYN can inhibit the E-cadherin protein expression, which is the crucial protein for epithelial-mesenchymal transformation (EMT) in tumor lymphatic metastasis (39). It is possible that HA oligosaccharides are responsible for the increased activation of LAYN in these tumors and can potentially enhance the secretion of inflammatory factors which leads to the recruitment of more immune cells in the tumor microenvironment (TME). Activation of LAYN expression causes significant enhancement of the secretion of many inflammatory factors, such as interleukin (IL)-8, CCL5, soluble intercellular adhesion molecules (sICAM), and complement5 (C5/C5a) (42). This is likely caused by tumor mediated immune cell recruitment by diverse chemokines that are secreted by tumor cells due to activation of relative signaling in the TME (43). Therefore, interactions between LAYN and HA oligosaccharides could be a potential mechanism for the correlation of LAYN expression with immune infiltration and poor prognosis in COAD and STAD.

In summary, increased LAYN expression correlates with poor prognosis and increased immune infiltration levels in CD8+ T cells, CD4+ T cells, macrophages, neutrophils and DCs of multiple cancers, especially in colon and gastric cancers. In addition, in gastic and colon cancers, LAYN expression potentially contributes to the regulation of tumor-associated macrophages (TAMs), DCs, T cell exhaustion, and Tregs. Therefore, LAYN likely plays an important role in immune cell infiltration and as a prognosis biomarker in patients with gastric and colon cancers.

## Author Contributions

JP and LR conceived the project and wrote the manuscript. HZ, SZ, JH, XZ, and HD participated in data analysis. LC participated in discussion and language editing. YP reviewed the manuscript.

### Conflict of Interest Statement

The authors declare that the research was conducted in the absence of any commercial or financial relationships that could be construed as a potential conflict of interest.
