# Supplementary material for: LAYN Is a Prognostic Biomarker and Correlated With Immune Infiltrates in Gastric and Colon Cancers
Source: Front Immunol. 2019 Jan 29;10:6. doi: 10.3389/fimmu.2019.00006 (PMC6362421; doi:10.3389/fimmu.2019.00006)
Supplement: Supplementary file 1 [file Data_Sheet_1.PDF]

Supplementary Materials

LAYN is a prognostic biomarker and correlated with immune infiltrates in gastric and colon cancers

Supplementary Table 1. LAYN expression in cancers verus normal tissue in oncomine database

Cancer	Cancer type	*P*-value	Fold change	Rank (%)	Sample	Reference (PMID)
Bladder	Superficial Bladder Cancer	2.40E-9	-1.634	6%	194	20421545
Breast	Invasive Breast Carcinoma Stroma	6.67E-14	6.031	7%	59	18438415
	Ductal Breast Carcinoma	1.54E-6	-4.758	5%	47	16473279
	Invasive Ductal Breast Carcinoma	5.26E-16	-2.019	13%	430	TCGA
	Invasive Breast Carcinoma	2.60E-9	-1.720	14%	137	TCGA
Colorectal	Rectal Adenocarcinoma	8.89E-14	1.883	12%	130	20725992
	Colon Carcinoma Epithelia	8.26E-5	1.659	14%	15	20957034
	Colorectal Carcinoma	4.83E-4	1.759	18%	60	20957034
	Colon Adenoma	6.45E-5	-2.009	8%	15	20957034
	Colon Adenoma	3.58E-4	-1.996	10%	57	18171984
	Colorectal Adenocarcinoma	5.94E-4	-1.568	14%	69	20957034
	Cecum Adenocarcinoma	1.55E-4	-2.033	28%	44	TCGA
	Rectal Adenocarcinoma	2.05E-4	-1.858	35%	82	TCGA
Gastric	Gastric Mixed Adenocarcinoma	4.66E-4	2.080	7%	35	19081245
Head and neck	Nasopharyngeal Carcinoma	2.40E-5	-1.877	2%	41	16912175
	Thyroid Gland Papillary Carcinoma	5.23E-5	-2.905	2%	18	16365291
Kidney	Clear Cell Renal Cell Carcinoma	2.59E-4	2.239	3%	18	14641932
	Clear Cell Renal Cell Carcinoma	5.27E-4	2.234	8%	31	19445733
Lung	Lung Adenocarcinoma	5.29E-25	-1.777	2%	116	22613842
	Lung Adenocarcinoma	2.45E-7	-2.071	2%	116	22613842
Lymphoma	Germinal Center B-Cell-Like Diffuse Large B-Cell Lymphoma	1.31E-6	2.19	3%	29	19412164
	Follicular Lymphoma	2.82E-18	2.189	3%	58	19412164
	Activated B-Cell-Like Diffuse Large B-Cell Lymphoma	3.51E-10	1.786	4%	37	19412164
	Diffuse Large B-Cell Lymphoma	1.68E-14	2.019	5%	64	19412164
	Hodgkin's Lymphoma	8.17E-4	3.017	12%	37	18794340
	Diffuse Large B-Cell Lymphoma	5.66E-4	1.558	15%	36	18794340
Ovarian	Ovarian Serous Adenocarcinoma	8.50E-9	-4.342	6%	53	19486012
Pancreas	Pancreatic Ductal Adenocarcinoma	5.91E-11	2.864	3%	78	19260470
Prostate	Prostate Carcinoma	1.62E-7	-1.708	4%	87	22722839
Others	Skin Basal Cell Carcinoma	2.69E-5	2.010	3%	19	18442402
	Teratoma, NOS	3.90E-6	2.206	10%	20	16424014
	Embryonal Carcinoma, NOS	2.30E-5	1.554	14%	21	16424014

Supplementary Table 2. Relation between LAYN expression and patient progonsis of different cancer in Prognoscan database.

Cancer type	Dataset	Endpoint	N	Hazard ratio (95%CI)	Cox *P*
Bladder cancer	GSE13507	OS	165	0.92 [0.65 - 1.29]	0.617
	GSE13507	DSS	165	1.19 [0.82 - 1.71]	0.361
Blood cancer	GSE12417-GPL97	OS	163	0.78 [0.14 - 4.47]	0.782
	GSE12417-GPL570	OS	79	2.52 [0.73 - 8.70]	0.144
	GSE16131-GPL97	OS	180	1.38 [0.79 - 2.41]	0.264
	GSE2658	DSS	559	1.03 [0.83 - 1.27]	0.807
Brain cancer	GSE4271-GPL97	OS	77	1.29 [0.84 - 1.98]	0.244
	GSE7696	OS	70	0.79 [0.55 - 1.13]	0.189
	GSE4412-GPL97	OS	74	1.67 [0.85 - 3.26]	0.137
	GSE16581	OS	67	2.13 [0.99 - 4.55]	0.053
Breast cancer	GSE19615	DMFS	115	0.40 [0.09 - 1.75]	0.222
	GSE12276	RFS	204	0.81 [0.64 - 1.03]	0.085
	GSE6532-GPL570	RFS	87	0.92 [0.59 - 1.44]	0.728
	GSE6532-GPL570	DMFS	87	0.92 [0.59 - 1.44]	0.728
	GSE9195	RFS	77	1.08 [0.41 - 2.85]	0.877
	GSE9195	DMFS	77	1.83 [0.59 - 5.64]	0.293
	GSE1456-GPL97	OS	159	0.41 [0.23 - 0.72]	0.002
	GSE1456-GPL97	RFS	159	0.57 [0.31 - 1.03]	0.061
	GSE1456-GPL97	DSS	159	0.48 [0.24 - 0.97]	0.04
	GSE3494-GPL97	DSS	236	0.68 [0.32 - 1.42]	0.306
	GSE4922-GPL97	DFS	249	0.84 [0.46 - 1.55]	0.585
Colorectal cancer	GSE17536	OS	177	1.50 [0.98 - 2.29]	0.06
	GSE17536	DSS	177	1.84 [1.15 - 2.97]	0.012
	GSE17536	DFS	145	2.32 [1.27 - 4.25]	0.006
	GSE14333	DFS	226	1.72 [1.21 - 2.46]	0.003
	GSE17537	OS	55	2.21 [0.93 - 5.27]	0.07
	GSE17537	DFS	55	2.06 [0.77 - 5.54]	0.152
	GSE17537	DSS	49	1.88 [0.63 - 5.63]	0.256
Eye cancer	GSE22138	DMFS	63	1.90 [1.08 - 3.35]	0.026
Head & neck cancer	GSE2837	RFS	28	0.44 [0.07 - 2.72]	0.379
Lung cancer	GSE13213	OS	117	0.75 [0.43 - 1.31]	0.31
	GSE13213	OS	117	1.01 [0.75 - 1.37]	0.933
	GSE31210	OS	204	1.86 [0.84 - 4.13]	0.126
	GSE31210	RFS	204	1.43 [0.79 - 2.60]	0.235
	GSE11117	OS	41	0.88 [0.46 - 1.68]	0.699
	GSE3141	OS	111	0.66 [0.44 - 1.00]	0.051
	GSE8894	RFS	138	0.94 [0.81 - 1.09]	0.383
	GSE17710	OS	56	1.06 [0.79 - 1.44]	0.692
	GSE17710	RFS	56	1.09 [0.82 - 1.46]	0.556
	GSE17710	RFS	56	1.11 [0.84 - 1.47]	0.465
	GSE17710	OS	56	1.02 [0.75 - 1.39]	0.882
Ovarian cancer	GSE9891	OS	278	1.40 [1.16 - 1.69]	<0.001
	GSE17260	PFS	110	1.22 [0.97 - 1.54]	0.095
	GSE17260	PFS	110	1.17 [0.67 - 2.06]	0.584
	GSE17260	OS	110	1.13 [0.84 - 1.53]	0.416
	GSE17260	OS	110	1.34 [0.62 - 2.88]	0.453
Skin cancer	GSE19234	OS	38	1.10 [0.72 - 1.69]	0.659

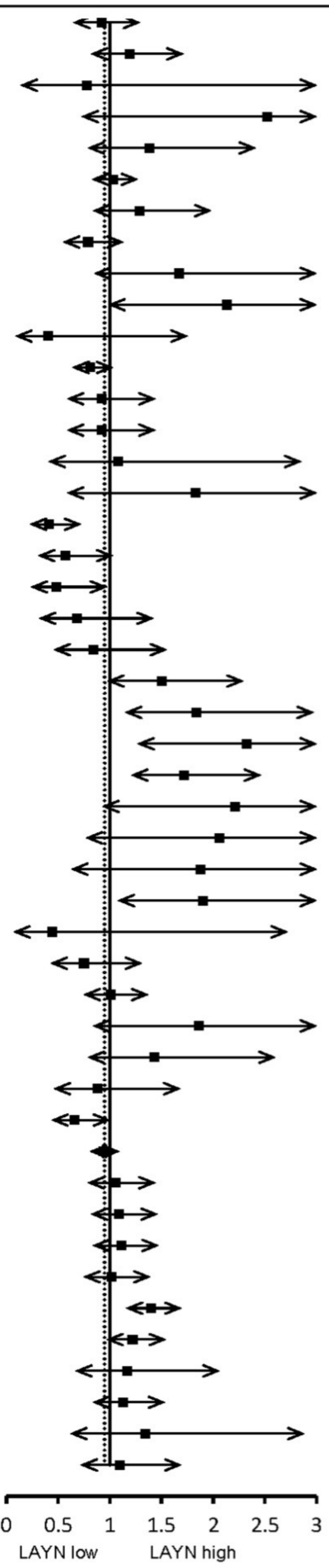

Supplementary Figure 1. Correlation of LAYN expression with prognostic values in diverse types of cancer.

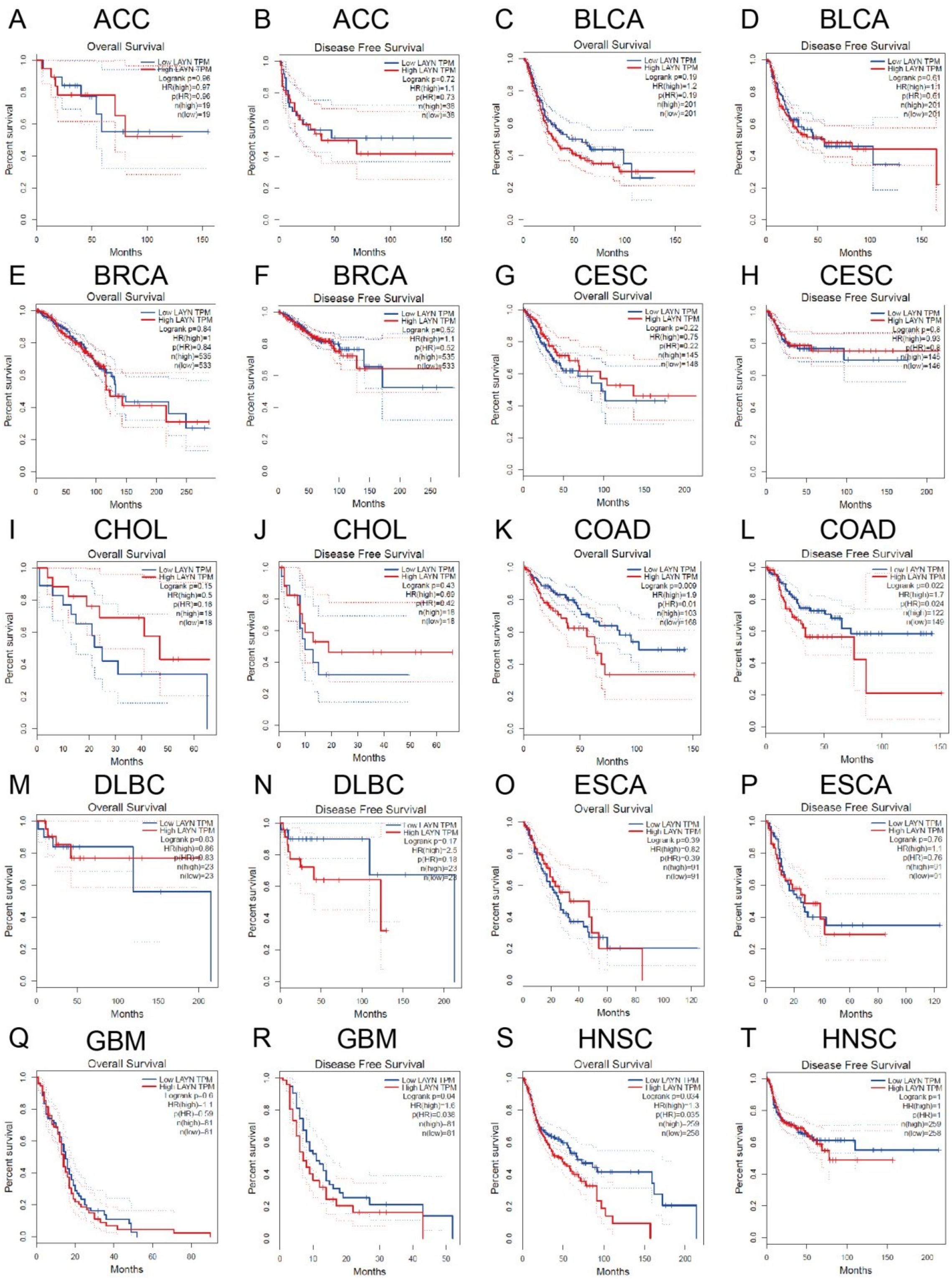

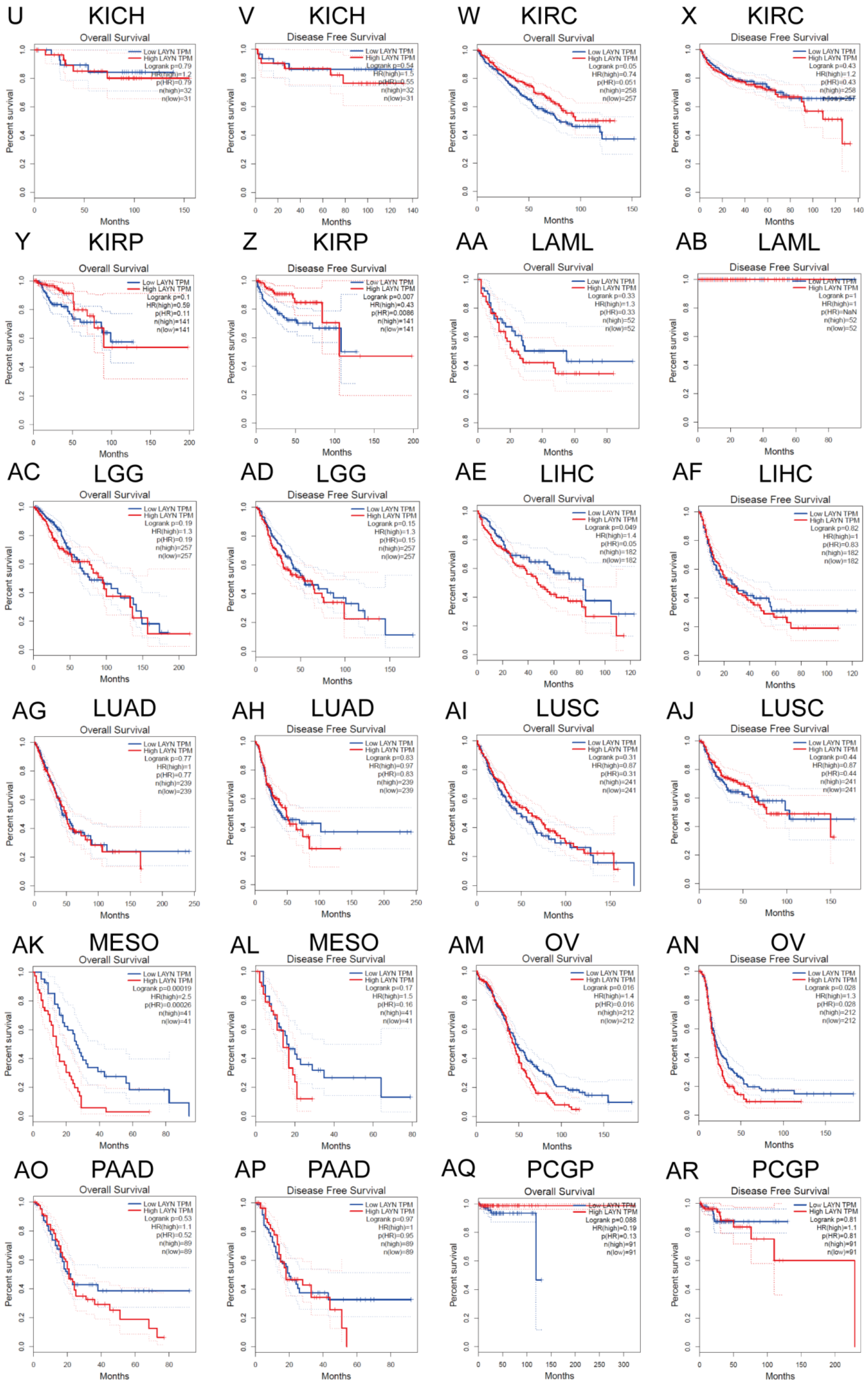

U KICH
Overall Survival
V KICH
Disease Free Survival
W KIRC
Overall Survival
X KIRC
Disease Free Survival
Y KIRP
Overall Survival
Z KIRP
Disease Free Survival
AA LAML
Overall Survival
AB LAML
Disease Free Survival
AC LGG
Overall Survival
AD LGG
Disease Free Survival
AE LIHC
Overall Survival
AF LIHC
Disease Free Survival
AG LUAD
Overall Survival
AH LUAD
Disease Free Survival
AI LUSC
Overall Survival
AJ LUSC
Disease Free Survival
AK MESO
Overall Survival
AL MESO
Disease Free Survival
AM OV
Overall Survival
AN OV
Disease Free Survival
AO PAAD
Overall Survival
AP PAAD
Disease Free Survival
AQ PCGP
Overall Survival
AR PCGP
Disease Free Survival
Low LAYN TPM
High LAYN TPM
Percent survival
Months

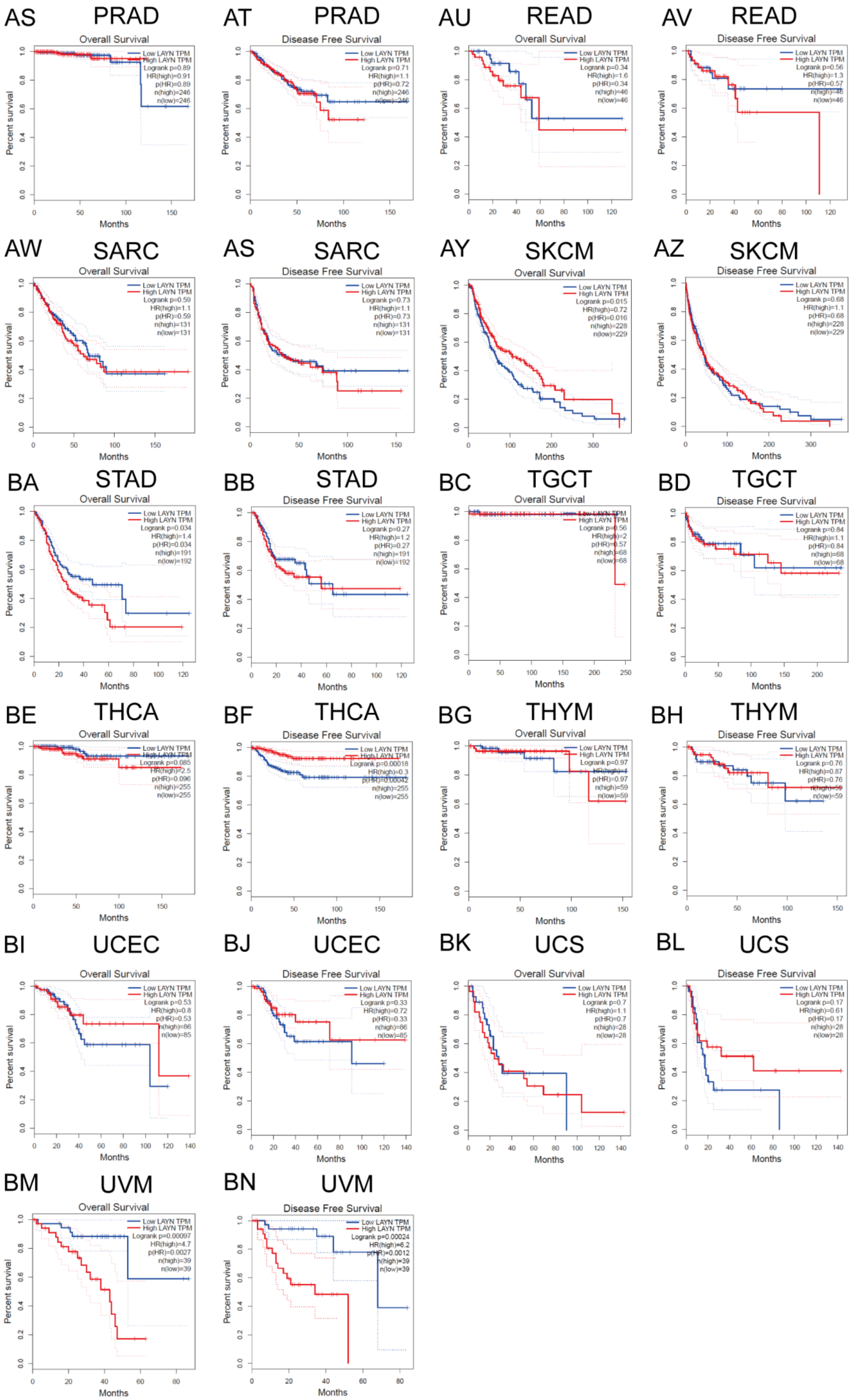
AS PRAD Overall Survival
AT PRAD Disease Free Survival
AU READ Overall Survival
AV READ Disease Free Survival
AW SARC Overall Survival
AS SARC Disease Free Survival
AY SKCM Overall Survival
AZ SKCM Disease Free Survival
BA STAD Overall Survival
BB STAD Disease Free Survival
BC TGCT Overall Survival
BD TGCT Disease Free Survival
BE THCA Overall Survival
BF THCA Disease Free Survival
BG THYM Overall Survival
BH THYM Disease Free Survival
BI UCEC Overall Survival
BJ UCEC Disease Free Survival
BK UCS Overall Survival
BL UCS Disease Free Survival
BM UVM Overall Survival
BN UVM Disease Free Survival
Low LAYN TPM
High LAYN TPM
Percent survival
Months

Supplementary Figure 1. Correlation of LAYN expression with prognostic values in diverse types of cancer. overall survival and disease free curves comparing the high and low expression of LAYN in Adrenocortical carcinoma(ACC) (A-B) , Bladder Urothelial Carcinoma (BLCA) (C-D), Breast invasive carcinoma (BRCA) (E-F), Cervical squamous cell carcinoma and endocervical adenocarcinoma(CESC) (G-H), Cholangio carcinoma(CHOL) (I-J), Colon adenocarcinoma(COAD) (K-L), Lymphoid Neoplasm Diffuse Large B-cell Lymphoma(DLBC) (M-N), Esophageal carcinoma(ESCA) (O-P), Glioblastoma multiforme(GBM) (Q-R) ,Head and Neck squamous cell carcinoma(HNSC) (S-T), Kidney Chromophobe(KICH) (U-V), Kidney renal clear cell carcinoma(KIRC) (W-X), Kidney renal papillary cell carcinoma(KIRP) (Y-Z), Acute Myeloid Leukemia(LAML) (AA-AB), Brain Lower Grade Glioma(LGG) (AC-AD), Liver hepatocellular carcinoma(LIHC) (AE-AF), Lung adenocarcinoma(LUAD) (AG-AH), Lung squamous cell carcinoma(LUSC) (AI-AJ), Mesothelioma(MESO) (AK-AL), Ovarian serous cystadenocarcinoma(OV) (AM-AN), Pancreatic adenocarcinoma(PAAD) (AO-AP), Pheochromocytoma and Paraganglioma(PCPG) (AQ-AR), Prostate adenocarcinoma(PRAD) (AS-AT), Rectum adenocarcinoma(READ) (AU-AV), Sarcoma(SARC) (AW-AX), Skin Cutaneous Melanoma(SKCM) (AY-AZ), Stomach adenocarcinoma(STAD) (BA-BB), Testicular Germ Cell Tumors(TGCT) (BC-BD), Thyroid carcinoma(THCA) (BE-BF), Thymoma(THYM) (BG-BH), Uterine Corpus Endometrial Carcinoma(UCEC) (BI-BJ), Uterine Carcinosarcoma(UCS) (BK-BL), Uveal Melanoma(UVM) (BM-BN).

Supplementary Figure 2. Correlation of LAYN expression with immune infiltration level in diverse type cancers via TIMER database.

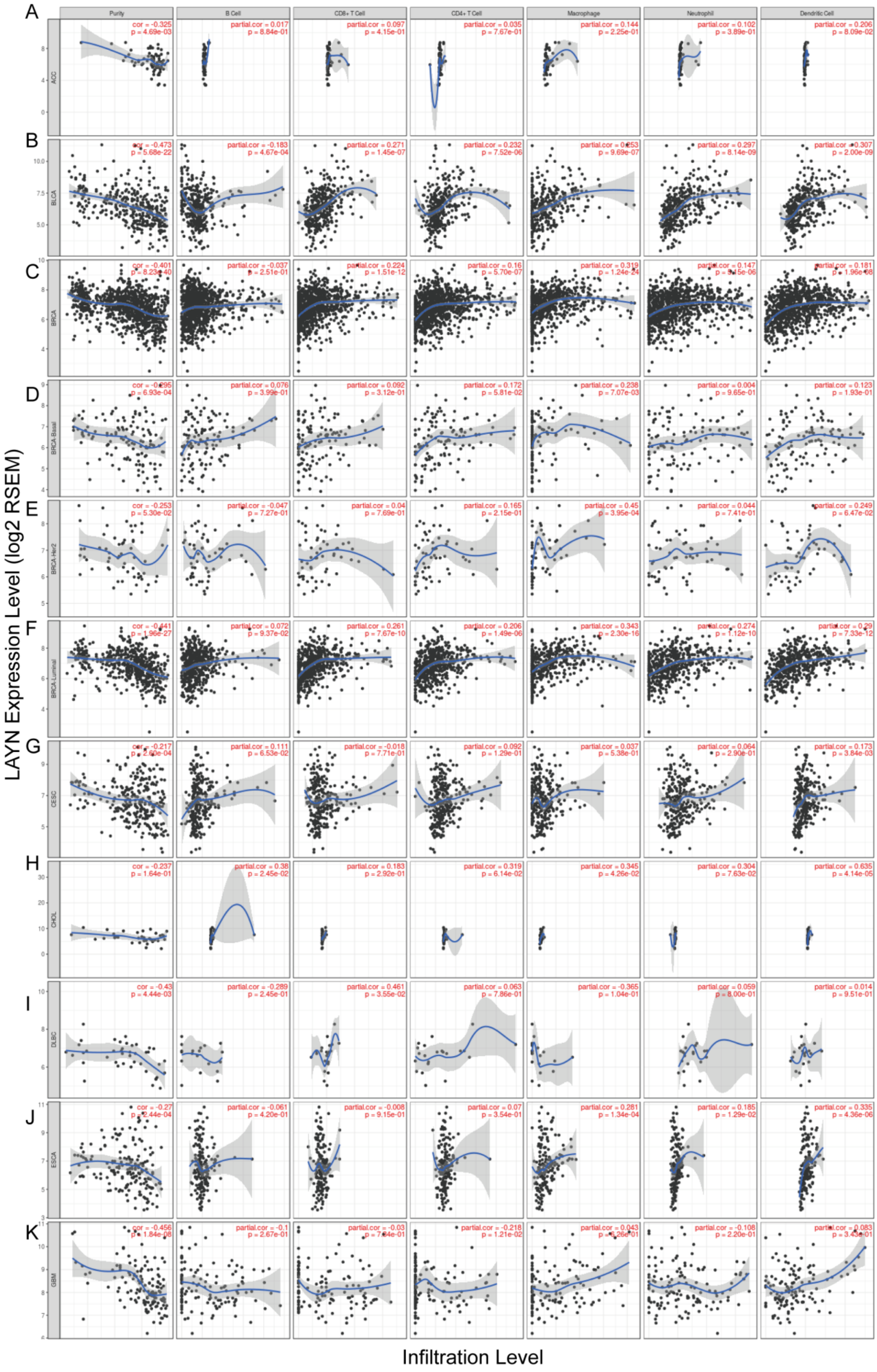

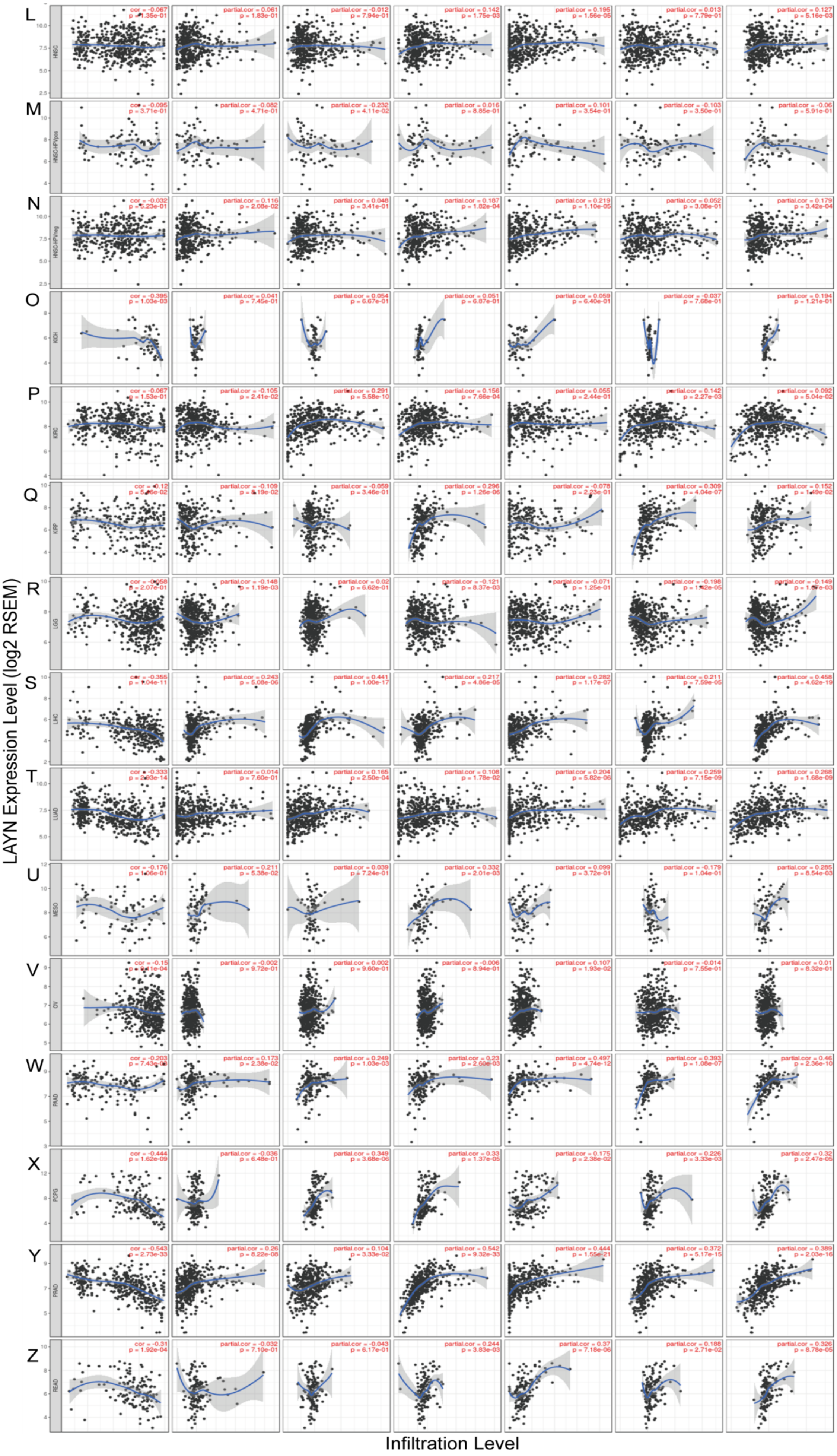

L
M
N
O
P
Q
R
S
T
U
V
W
X
Y
Z
HNSC
HNSC-HPVpos
HNSC-HPVneg
KICH
KIRC
KIRP
LGG
LIHC
LUAD
MESO
OV
PAAD
PCPG
PRAD
READ
LAYN Expression Level (log2 RSEM)
Infiltration Level

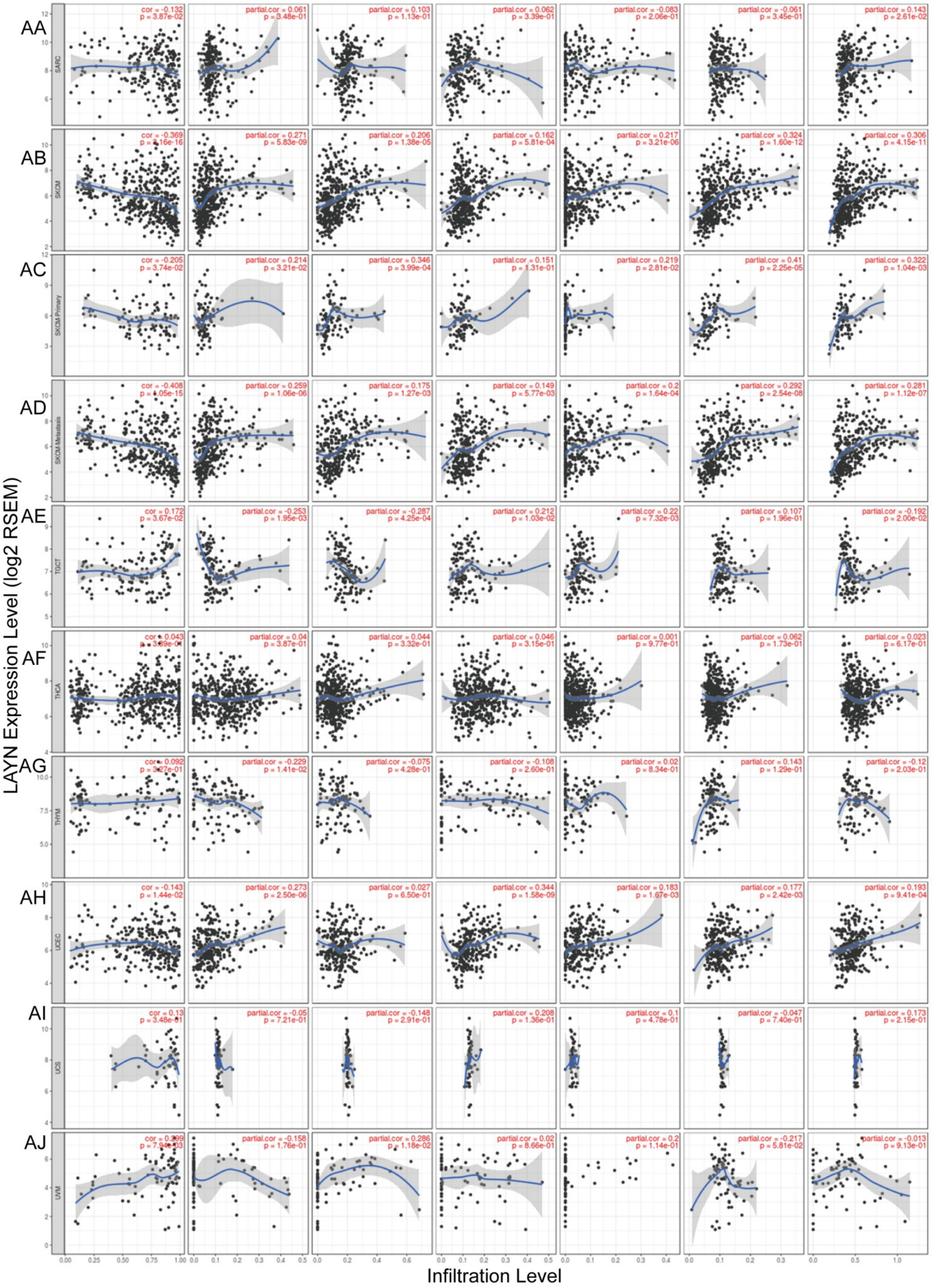

Supplementary Figure 2. Correlation of LAYN expression with immune infiltration levels in Adrenocortical carcinoma(ACC) (A) , Bladder Urothelial Carcinoma (BLCA) (B), Breast invasive carcinoma (BRCA) (C),

Breast invasive carcinoma-Basal (BRCA-Basal) (D), Breast invasive carcinoma-Her2 (BRCA-Her2) (E), Breast invasive carcinoma-Luminal (BRCA- Luminal) (F), Cervical squamous cell carcinoma and endocervical adenocarcinoma(CESC) (G), Cholangio carcinoma(CHOL) (H), Lymphoid Neoplasm Diffuse Large B-cell Lymphoma(DLBC) (I), Esophageal carcinoma(ESCA) (J), Glioblastoma multiforme(GBM) (K) ,Head and Neck squamous cell carcinoma(HNSC) (L), Head and Neck squamous cell carcinoma-HPVpos(HNSC-HPVpos) (M) , Head and Neck squamous cell carcinoma-HPVneg(HNSC-HPVneg) (N), Kidney Chromophobe(KICH) (O), Kidney renal clear cell carcinoma(KIRC) (P), Kidney renal papillary cell carcinoma(KIRP) (Q), Brain Lower Grade Glioma(LGG) (R), Liver hepatocellular carcinoma(LIHC) (S), Lung adenocarcinoma(LUAD) (T), Mesothelioma(MESO) (U), Ovarian serous cystadenocarcinoma(OV) (V), Pancreatic adenocarcinoma(PAAD) (W), Pheochromocytoma and Paraganglioma(PCPG) (X), Prostate adenocarcinoma(PRAD) (Y), Rectum adenocarcinoma(READ) (Z), Sarcoma(SARC) (AA), Skin Cutaneous Melanoma(SKCM) (AB), Skin Cutaneous Melanoma-Primary(SKCM- Primary) (AC), Skin Cutaneous Melanoma-Metastasis(SKCM- Metastasis) (AD), Testicular Germ Cell Tumors(TGCT) (AE), Thyroid carcinoma(THCA) (AF), Thymoma(THYM) (AG), Uterine Corpus Endometrial Carcinoma(UCEC) (AH), Uterine Carcinosarcoma(UCS) (AI), Uveal Melanoma(UVM) (AJ).
